# Problems and Barriers Regarding the Admission, Financing, and Service Provision of Digital Health Apps: Qualitative Stakeholder Survey

**DOI:** 10.2196/73332

**Published:** 2026-02-09

**Authors:** Felix Plescher, Godwin Denk Giebel, Carina Abels, Silke Neusser, Bonnie Kampka, Kirstin Börchers, Jürgen Wasem, Nikola Blase

**Affiliations:** 1 Faculty of Economics, Institute for Healthcare Management and Research, University Duisburg-Essen, Thea-Leymann-Street 9, Essen, Germany, 49 2011832912; 2 QM Börchers Consulting +, Herne, Germany

**Keywords:** mobile health apps, mHealth, stakeholder, DiGA, digitale Gesundheitsanwendungen, digital health apps, challenges, digital health care, health innovation, regulation, mobile health

## Abstract

**Background:**

Since their introduction with the Digital Care Act in 2019, selected digital health apps (DiGA) have been a part of the German statutory health care system. In order to become a DiGA, digital health apps have to complete a certification process covering both technical and evidence-related aspects. After completion, DiGA are added to the DiGA directory, containing a list of all reimbursable DiGA within German statutory health insurance. The first apps were added at the end of 2020, with the number steadily increasing. The novelty of this digital health care service and the fast implementation led to problems and barriers to optimal use along the way, which are studied from different stakeholder perspectives in this paper.

**Objective:**

The aim of this survey was to identify problems and barriers in the context of the admission, financing, and service provision of DiGA in the statutory health care system in Germany.

**Methods:**

We used semistructured expert interviews to evaluate the perspective of stakeholders of the German health care system on DiGA. The interview guide was developed according to Helfferich. The interviews were transcribed and analyzed using the qualitative content approach by Mayring, with the adjustments by Kuckartz. We conducted web-based interviews with stakeholders between February and June 2022. The stakeholder collective consisted of DiGA, statutory health insurance, physician, patient, technological, and quality assurance representatives.

**Results:**

To identify problems from stakeholder perspectives regarding the admission, financing, and service distribution of DiGA, 21 interviews were conducted. The interviewed stakeholders reported problems with the authorization of DiGA and the corresponding process, for example, the duration of the DiGA Fast Track process. DiGA prices and the different negotiation positions were criticized, and financial challenges for smaller DiGA manufacturers were noted. Another problem was seen in the reimbursement of DiGA, independent of actual use by the patients. Within service provision, the participants reported superordinate aspects, for example, the negative public perception of DiGA and negative statements from stakeholders. In relation to the direct care process, technical problems (eg, with activation codes or software surrounding DiGA prescription) and insufficient knowledge and skills on the side of the patients, as well as the medical providers, were mentioned.

**Conclusions:**

Digital health apps have the potential to improve health care by addressing health problems in new, innovative ways. Since the evidence-based and regulated use of this technology is relatively new, problems and barriers limiting the optimized, patient-centered use arose throughout the first years. This study provides an overview of problems and barriers in the context of DiGA in Germany from different stakeholder perspectives. Nevertheless, with these problems being continuously addressed, digital health apps are trending toward becoming a contributing factor to health care in Germany. Since other countries showed interest in implementing a federally regulated approach similar to Germany, valuable implications can be drawn from this survey.

## Introduction

The inclusion and adoption of digitalized processes, products, and the integration and development of digital health care infrastructure is an ongoing process throughout health care systems around the world. Within the German health care system, the transition from analog to digital, connected structures is facing difficulties and still needs improvement (eg, [1,2]). For example, Germany is lagging behind other countries in the process of implementing an electronic health record [2]. Some solutions, for example, video consultation for statutory health care providers, were used frequently after implementation, though this might be partly due to contact restrictions during the COVID-19 pandemic. Other initiatives, like the above-mentioned electronic health record (ePA), are still in evolving stages [3].

German health legislation emphasized the focus on a more digitalized and connected health care system with the introduction of the Digital Healthcare Act. As part of this legislative agenda, selected digital health apps (DiGA) were added as health care services for statutorily insured people. Since the introduction in 2019, DiGA are reimbursable within the German statutory health insurance (SHI) system. As DiGA can be both mobile apps and web-based interventions, the term digital health apps also incorporates both these options. In general, after the completion of a certification process (DiGA Fast-Track process), DiGA are added to a directory, which lists all reimbursable apps. Once DiGA are listed, they are eligible for use within the German SHI system [4]. Over a year after the legislation was passed, the first DiGA were added to the DiGA directory in September 2020 [5]. There are a number of requirements for eligibility for reimbursement within the German SHI system. All listed applications need to be certified as medical devices according to the European Medical Device Regulation [6]. Furthermore, the listing requires the additional completion of a certification process, which is carried out by a federal institute (Federal Institute for Drugs and Medical Devices [BfArM: Bundesinstitut für Arzneimittel und Medizinprodukte]) covering both general criteria and a positive medical effect for the patients. The general requirements include safety, functionality, quality, as well as data security and protection [7]. The positive medical effect can either be proven with medical benefit, for example, improvement of the health status, or patient-relevant improvement of structures and processes, for example, adherence [8].

There are 2 different alternatives when applying for a listing in the DiGA directory, which have to be declared when starting the application process. All apps applying for a listing have to fulfill the general requirements, as described above. If the positive care effect has been proven in studies complying with the study requirements (§ 10 Digital Health Applications Regulation), apps can apply for a permanent listing. Nevertheless, if there is not enough evidence yet to prove the positive care effect, a preliminary listing for a period of up to 12 months (with a possible extension of 12 months) can be obtained. Systematic data evaluations indicating positive (medical) effects and an evaluation concept have to be submitted. The applying DiGA manufacturer has to prove the medical benefit within a period of up to 12 months, with a subsequent listing if successful [8].

Starting with the implementation of the DiGA directory, a total number of 71 DiGA have been listed (as of July 2025) so far. A total of 14 DiGA have been removed from the DiGA directory, leaving 57 currently listed. About 20% (13 of 57 currently listed) of DiGA are preliminarily added to the DiGA directory, with the final proof of a positive care effect still pending. The remaining 44 have proven the positive care effect through scientific studies and are therefore permanently listed. DiGA for mental diseases currently account for the largest share with almost 50% (28/57, 49%). Endocrine, nutritional, and metabolic diseases, diseases of the musculoskeletal systems, and diseases of the nervous system are other examples of indications that currently have multiple DiGA listed [9].

After successful completion of the certification process, manufacturers can set a price they consider suitable for the first 12 months. If DiGA for comparable indications are already added to the DiGA directory, the price cannot exceed the group price. The group price is calculated for DiGA of similar indications in order to ensure equal pricing [10]. After this, a price is negotiated between the National Association of Statutory Health Insurance Funds (GKV-SV: GKV-Spitzenverband) and the DiGA manufacturer, with repayments if the negotiated price is lower than initially set [11].

In order to have the cost reimbursed by the SHI funds, patients have 2 options. They can receive the application if it is prescribed by a physician or directly from the SHI fund without prior prescription (documented diagnosis, eg, in patients’ electronic health record within last 6 mo [12]). Both options are fully reimbursed through SHI [13]. Giebel et al [14] described DiGA care and the corresponding processes in detail.

Patients obtain access to the respective apps through activation codes. About 81% of all activation codes have actually been used. The number of used activation codes increased from 50,122 DiGA in 2021 to 374,377 DiGA in total through 2023 to 861,053 at the end of 2024. But not all DiGA are used at the same rate. The top 6 DiGA account for almost two-thirds of the entered activation codes (approximately 500,000). This includes DiGA for metabolic diseases, diseases of the musculoskeletal system, diseases of the ear, as well as mental and behavioral diseases [15,16]. There are also differences when looking at the distribution of DiGA across indications. DiGA for mental diseases accounts for the largest share, with about 259,000 prescriptions. Metabolic diseases make up about 241,000 prescriptions, and diseases of the musculoskeletal system around 137,000 [16].

The introduction and corresponding processes of DiGA were a new and challenging task for the German health care system. As part of a larger research project (Refer to Acknowledgements), which is funded by the Federal Joint Committee (Gemeinsamer Bundesausschuss; Funding code: 01VSF20007), the presented qualitative analysis identified problems and barriers of DiGA regarding the regulation and organization, as well as the use in everyday practice. Even with increasing prescription numbers since the introduction of DiGA, skeptical public statements by different stakeholders remain [17,18] (see Results for more), showing there is still disagreement about the disruption and success of DiGA. This poses the question of which problems are still persisting concerning DiGA and their corresponding processes.

With DiGA being evaluated for a lot of aspects prior to admission, we adjusted the aim of the study from DiGA itself—for example, problems with the technical components of DiGA—to certification, financing, and use of the same. Furthermore, since just a few countries have actually implemented digital health apps (similar to DiGA) in their health care system, we decided to focus on problems and barriers from the German infrastructural health care system perspective.

Prior research by Giebel et al [1] described problems with implementation of digital health apps “because of a lack of infrastructure […], [l]ack of access and organizational barriers, such as lack of preparedness of health care systems and reimbursement structures.” A review investigating mHealth (mobile health) interventions during the COVID-19 pandemic identified barriers such as insufficient interaction, time, or privacy, but also the nonacademic nature of some apps [19]. This shows that there are studies investigating problems surrounding digital health apps themselves, yet they often include a wide range of interventions, potentially limiting the transferability of the generated results. At the same time—to the best of our knowledge—the perspective of a large collective of stakeholders concerning the process and infrastructure around federally regulated digital health apps has not had an exclusive focus in the existing literature. This is particularly interesting in the context of German DiGA, as different stakeholder groups with competing interests (eg, [20,21]) are involved with the implementation of this new federal health care service. Previous qualitative research in Germany covering DiGA has, for example, focused on certain aspects in a specific medical field (eg, [therapeutic] suitability in rheumatology) [22] or the perspective of a single stakeholder group (eg, physicians, patients) [23,24]. In addition, an article addressing a similar stakeholder group [25] has used a broader focus covering a wide range of aspects of DiGA (eg, potentials of DiGA for patients, suggestions for the improvement of DiGA care).

Our study is adding to current literature by focusing on digital health apps from an infrastructural standpoint. As described above, other studies have also mentioned infrastructural problems and barriers. However, these studies, for example, include external mHealth apps and therefore issues with the implementation into a health care system [1,26] or a broader scope [25]. Our approach covers mHealth apps, which have been implemented as a health care service in a statutory health care system, and the corresponding infrastructural issues from a stakeholder perspective. These include DiGA, SHI, physician, patient, technological, and quality assurance representatives, as well as experts from federal institutions. The stakeholder perspective is also important, as other research has suggested that more stakeholder collaboration is necessary in order to realize the full potential of mHealth apps [26].

This survey is being conducted shortly after the first DiGA were implemented; therefore, it will possibly identify first-hand problems during the implementation process. To provide perspective on how the DiGA market has evolved, the following paper also lays out what adaptations have been made to solve them and what problems may still persist. This study contributes to digital health app research by giving valuable insights for health care policy makers aiming to implement a system similar to the German DiGA approach.

## Methods

### Overview

The qualitative stakeholder survey was planned, conducted, and the results were reported in accordance with the Consolidated Criteria for Reporting Qualitative Studies (COREQ) checklist (refer to Checklist 1) [27]. We identified the perspective of the stakeholders using semistructured interviews. The study consisted of 3 main stages: In the first stage, the interviews were prepared by recruiting participants and developing an interview guide. The second stage included the actual interviews. During the third stage, interviews were transcribed, and data were analyzed and reported as presented below.

### Ethical Considerations

The study was presented to and approved to be in accordance with the current Ethics Regulation by the Ethics Committee of the Medical Faculty of the University Duisburg-Essen (no conventional ethics approval vote was necessary). Additionally, the suitability of the data for publication was approved by the Ethics Committee of the Faculty of Economics of the University Duisburg-Essen.

An agreement covering the terms and conditions of participation and data protection was sent to potential candidates beforehand. All stakeholders had to sign the agreement before participating in the study (refer to the Recruitment section).

The interviews were recorded and pseudonymized during the subsequent transcription process. This ensured that transcripts did not contain any identifying information and the statements made cannot be traced back to the respective person (refer to the Interviews and Transcription sections).

Participants did not receive any compensation for participation in the study.

### Recruitment

An initial list of stakeholders was assembled during the application for project funding in 2020 (see Acknowledgments for more). Before starting the recruitment process, this list was reviewed, and necessary adaptations were made. These adaptations were based on the DiGA legislation, which was not available during project application [7,8,13,15,16]. The documents were checked for additional stakeholder groups, and new, not yet included ones were added. The final list included the association of SHI physicians, representatives of patient organizations, the SHI funds, digital health app companies, and experts in the field of quality management, data security and protection, as well as medical device regulation. The recruitment process started in December 2021 and continued until May 2022. A total of 23 organizations were contacted. After each interview, the research team decided if sufficient information for the stakeholder group (in reliance on their involvement in DiGA processes) was collected. Once saturation was reached for the stakeholder group, no further requests were sent out. However, if stakeholders, who were contacted before, decided to participate in the study, they were also included. Patients (representatives) were only represented by a small subsample, even though they are of particular importance in the context of DiGA, because we did an additional interview and focus group study prior to this study, exclusively covering patients’ perspectives [28].

We approached the representatives of the organizations by direct phone call or e-mail. We requested the participation of the employee responsible for quality assurance, if available. If participation in the study was agreed, additional information about the topics of the discussion and the terms and conditions of participation and data protection was transmitted. We provided information on DiGA and the research project in order to ensure all participants had the same base knowledge before taking part in the interviews. Nonresponders to the telephone call were followed up by e-mail (directly after the phone call) containing the aforementioned information and additional general information about the study. An additional follow-up was sent out one week after the initial phone call. Participants had to sign the agreement about participation and data protection before the start of the interview.

### Interview Guide

The interview guide was developed according to Helfferich et al [29]. We used a semistructured interview guide to provide guidance and cover the relevant topics, but also leave room to address aspects not included in the interview guide. As the interviews are part of a larger research project, the interview guide was based on results generated earlier in the project. A scoping review [1] covering general problems and barriers related to digital health apps provided the evidence base for the main topics. This was extended using results from a—as mentioned above—qualitative survey with DiGA users and nonusers [28]. As the previous study modules focused on the apps themselves, the interview guide aimed at identifying problems and barriers concerning the infrastructure and process surrounding DiGA. Open questions were used to identify aspects without influencing the interview participants. More precise follow-up questions were asked to inquire more deeply about certain aspects. The interview guide was not available to the study participants prior to the interviews. Only the topics of the interview guide were communicated. We carried out a pretest interview and made adaptations to the interview guide in order to improve comprehensibility and feasibility. As the following interviews were conducted within the planned time frame and the interview partners did not have problems answering the question, the adapted interview guide was used throughout the remaining study.

### Interviews

The interviews were conducted by 4 of the authors of this article (NB, CA, GDG, and FP) between February and June 2022. Two of the interviewers are female and 2 male, with 2 senior researchers (PhD: Dr med/Dr rer medic) and 2 junior researchers (MSc/MA). All interviewers are part of the QuaSiApps project team (NB is project lead), with prior experience in qualitative research projects. During the interviews, only the interview study participants and part of the research team were present. There were no prior relationships between the researchers and any of the study participants. Participants did not receive any compensation for participation in the study.

The interviews were carried out using the videoconference platform ZOOM (Zoom Video Communications Inc). One of the researchers moderated the conversation with at least one additional researcher taking notes on the main talking points and overseeing the technical infrastructure. The interview sessions were recorded in accordance with the signed agreement using the integrated recording function of ZOOM. An additional disclaimer was stated by the moderator before starting the recording. The duration ranged from about 50 minutes to 1 hour and 45 minutes.

### Transcription

After the conclusion, the recorded interviews were transcribed following the transcription rules by Claussen et al [30]. The transcript was revised by an independent reviewer from the research team, not involved in the transcription. After discussing the remarks and correcting mistakes, the transcript was finalized. All individuals taking part in the interviews, as well as organizations and institutions, were pseudonymized. Therefore, no connections can be made between the statements made and the participants.

### Analysis

We analyzed the interviews in accordance with the qualitative content analysis approach by Mayring and the extensions by Kuckartz (content-structuring qualitative analysis) [31,32]. The analysis was carried out as described below using MAXQDA (VERBI), a software tool for qualitative research [33]. The transcripts were imported into MAXQDA, and a coding system containing deductive and inductive codes served as a tool for content extraction. The main topics of the interview guide provided the deductive codes. These included the codes “Problems: Admission,” “Problems: Financing” and “Problems: Service Provision,” which represent the primary outcomes of our study. We also added deductive codes covering problems regarding the main stakeholders in the context of DiGA, for example, DiGA manufacturers, SHI funds, or patients. Throughout the first coding process, inductive codes were added to the code system. The full coding scheme was reviewed, discussed, and finalized by the research team (Multimedia Appendix 1). After completion of the first coding process and the finalization of the coding scheme, all interview transcripts were coded again using the final code system, which included all inductive and deductive codes. This process was also carried out by 2 researchers independently, with a third researcher reviewing coded transcripts and deciding in case of disagreement. The codes were further grouped into the 3 categories: admission, financing, and service provision. This step was reviewed, discussed, and necessary adaptations were made by the research team. The results of the analysis and corresponding quotes were sent to the study participants for review. We did not receive any additional comments or requests for corrections.

## Results

### Interview Sample

Of the 23 contacted stakeholders, 2 declined and 21 agreed to participate in the study. None of the 21 stakeholders dropped out after further information about the study and the terms and conditions were sent out. The interviews were conducted with all 21 stakeholders. None of the stakeholders decided to withdraw after the interviews were completed. The characteristics of the 21 included participants are presented in Table 1.

**Table 1.** Surveyed stakeholder groups.

Stakeholder group	Participants, n (%)
DiGA representatives^a^	7 (33.3)
SHI representatives^b^	6 (28.6)
Physician representatives	2 (9.5)
Patient representatives	1 (4.8)
Representative from the Federal Joint Committee	1 (4.8)
Technological experts	2 (9.5)
Quality assurance experts	2 (9.5)

a DiGA : Digitale Gesundheitsanwendung (digital health application).

b SHI: statutory health insurance.

### Interview Results

The results are presented for each of the 3 subtopics: admission, financing, and service provision. Anonymous quotes from the interviews are used to illustrate the mentioned aspects. In order to guarantee that, only the stakeholder group (eg, DiGA manufacturer) is mentioned. If not already described in the introduction, we provided additional context throughout the Results section about the specifications of the German DiGA systems in order to ensure a more comprehensive understanding of the presented problems and barriers.

As we aimed to investigate problems and barriers, we also wanted to mention that a large share of stakeholders expressed a generally positive attitude toward DiGA and DiGA processes during the early implementation phase. Better access to health care services for patients in rural regions (patient representative), bridging long waiting times (DiGA representative), and supporting general care processes (SHI representatives) are a few examples of potential benefits of DiGA, which were suggested during the interviews.

### Admission

Even though the fast process for digital health apps to become DiGA was generally seen as a good innovation, different stakeholders mentioned problems in the way the DiGA Fast-Track is structured and the corresponding responsibilities. Especially the DiGA manufacturers expressed that there is not enough expertise to evaluate—in particular—the technical, nonevidence-related aspects.

*So, the BfArM review is very focused on the whole topic of clinical evidence, e.g., study planning. That’s their main task, you don't have to talk about it. Yes, so I think in the area of usability, user-friendliness, that could be-, so that’s not their topic either. But I don't think it would do it any harm to go into it in more depth […].*

[DiGA manufacturer]

Other problems were seen in the high volume of applications for the BfArM and the low level of flexibility (DiGA manufacturer), which was partly credited to the new and challenging nature of implementing a new digital health solution (SHI representative).

There was also criticism about the duration of the DiGA Fast-Track. Upon application by the DiGA manufacturer, a decision has to be made by the BfArM within 3 months [34]. The shift away from the regular admission process of medical products into SHI (quality assurance stakeholder) and the relatively short certification process raised concerns about the quality of the assessment (quality assurance stakeholder, SHI representative, physician representatives).

*Because the assessment process has been rammed out of the ground by the legislator in a relatively short time, because it all wanted to happen very quickly and that is also - the terminology speaks for itself, fast-track procedure, eg, the fastest possible market entry is in our view a bit in the foreground and then naturally the proof of benefit comes a bit short. So that would be a structural problem or a very fundamental problem, which we still see.*

[Physician representative]

A similar aspect with a shifted focus was mentioned by DiGA manufacturers. They criticized that the 3-month assessment period is not put on hold when, after all initial paperwork is turned in, the BfArM requires additional paperwork from the applying DiGA company. This led to very short deadlines to process, produce, and turn in the required documents (DiGA manufacturer).

A diverse range of perspectives was reported surrounding the authorization requirements for both general and evidence-related aspects. On the one hand, the requirements were seen as too low (SHI representative); on the other hand, the steadily increasing requirements were described as limiting for the flexibility of innovation (DiGA manufacturers).

Since DiGA must be medical products and therefore are/were subject to external certification when obtaining the CE certification, problems surrounding the notified bodies (German: “Benannte Stellen,” eg, Technical Inspection Association) were also mentioned. Especially in the early days of DiGA certification, the insufficient levels of knowledge about DiGA within these companies and the low availability of appointments for certification were described (certification companies, DiGA manufacturer):

*And there are extreme waiting times and a kind of undersupply. So many people have a problem getting appointments at the designated places [certification companies]. So, there’s already a big bottleneck and you have to look at how realistic it is to implement it.*

[DiGA manufacturer]

There was also criticism concerning the innovation of the preliminary approved DiGA. Physician representatives admonished that the possibility of preliminary approved DiGA yields apps with low evidence to be reimbursed by SHI funds. The equivalent handling of preliminary and permanent approval may also distort the level of evidence between different DiGA (physician representative, SHI representative, DiGA manufacturer).

*Yes, what is also problematic, for example, is the fact that there are two stages. On the one hand, there is the stage where you are finally included in the BfArM directory and on the other hand, there is the stage where you are temporarily included and then have to provide evidence.*

[SHI representative]

*What is viewed negatively, at least from my point of view in terms of quality, is that we have provisional approval that is not based on RCTs, which has a certain risk because it suggests that these products without evidence have the same efficacy as products with evidence and that is of course a problem, especially for the medical profession, which we have been trying to push into evidence-based medicine for 20 years.*

[DiGA manufacturer]

A problem described by DiGA manufacturers regarding the conclusion of the Fast-Track process was the inconsistent handling of the market access. One manufacturer reported that after successfully becoming a DiGA, the first prescriptions are possible the next day, but in their case, no listing in the prescription software led to no prescriptions within the first days. A different manufacturer reported the same problem with no possibility of prescriptions due to the unavailability of a necessary interface port to the prescription software (DiGA manufacturer).

### Financing

The interviewed stakeholders mostly talked about the price of DiGA, the process of negotiation between the SHI funds and the DiGA manufacturer, and the price-related policy. DiGA manufacturers said the price expectations of health insurance funds indirectly show low willingness to negotiate and lead to high initial prices (DiGA manufacturer).

*The offers that the GKV-SV distributes here in the market mean that manufacturers anticipate that they will take the highest possible price at the time when it is possible, so that they can somehow reduce some of the debt that they have accumulated.*

[DiGA manufacturer]

They also criticized the superior negotiation position of the GKV-SV—the head organization of all SHI funds in Germany, since they get to deal with multiple DiGA manufacturers, but a single DiGA manufacturer only gets to deal with the GKV-SV once (DiGA manufacturer). One health insurance fund representative mentioned that—even with this negotiation position—they have difficulties achieving favorable SHI positions in price negotiations (SHI representative).

Multiple stakeholders took a look at the finance aspect from the DiGA manufacturers’ view and laid out that the financing of the app and corresponding activities (eg, evaluation studies, personnel) are challenging, especially for small companies (DiGA manufacturer, technological expert, patient representative).

*However, due to the hurdles posed by the legislator, it is not always easy for start-ups to raise the basic capital to gain a foothold in the market, especially when it comes to financing.*

[Technological expert]

One SHI representative mentioned that the refund of excess payments after price negotiations might lead to a market shrinkage, due to the superior financial resources of large companies compared with smaller DiGA manufacturers. Additionally, besides general criticism, a separate regulation for preliminary and permanently listed DiGA was called for, as the described regulation above is currently in place regardless of admission status. The necessity of usage and quality-related pricing was also pointed out (SHI representative).

As of now, DiGA are being reimbursed as soon as the patient downloads the app and enters the DiGA activation code, provided by the health insurance fund. Therefore, reimbursement is independent of usage. There were concerns among SHI representatives about the financing of DiGA without knowing if the app is actually used by the patient.

*Problems are certainly, as an initial survey of insured persons by the health insurance fund [name anonymized] has also shown, problems are certainly that you don't know whether and how the insured person uses this DiGA in the end.*

[SHI representatives]

### Service Provision

In the early days of DiGA, there was disagreement among the questioned stakeholders about how well the communication between different stakeholders was going. Especially, the manufacturers emphasized that negative public reporting does not help DiGA flourish. According to them, different organizations made negative remarks about DiGA or even gave out “warnings” (DiGA manufacturer). General skepticism among stakeholders when it comes to digitalization may also have an impact on how well DiGA are perceived. Multiple DiGA manufacturers added that this was accompanied by negative public statements and comments by SHI representatives about DiGA, which further hindered the development.

*I often see very sweeping arguments being used when DiGA are criticized. Often the basics are not so well known, the legal and that is of course-, so DiGA are sometimes also seen as a symbol for digitization per se. And then perhaps no distinction is made as to whether it is a project such as an e-prescription or a DiGA, but it is simply a blanket term for digitization and there is a great deal of skepticism*

[DiGA manufacturer]

The DiGA directory and the lack of (technical) information presented in it were also criticized among stakeholders. SHI and physician representatives both reported that they did not, or only in a limited fashion, receive access to selected DiGA (SHI representative, physician representative). The DiGA directory could not fill this knowledge gap sufficiently either, as especially SHI representatives pointed out that information about DiGA was too complex and no information on the quality of the applications was provided:

*We would say that the issue of transparency, or rather the lack of transparency, is a major problem from our point of view. Also, in terms of the lack of comparison options for the apps. We are of the opinion - at the moment, the DiGA directory, the BfArM directory is basically the source that you can look at. […] In other words, to prepare it differently for patients and physicians. We still see a lot of room for improvement, especially as it is currently the case that - when you look at the DiGA directory, you can't really find anything about quality at first glance.*

[SHI representatives]

Low cooperation was also seen in the work of medical software companies (German: “Praxisverwaltungssoftware [PVS] – Hersteller,” PVS manufacturer) responsible for technical solutions in SHI medical practices. This showed in slow adoption of existing practice software (Technological expert), establishment of own structures for app prescriptions (DiGA manufacturer), and little interest and knowledge in new DiGA policies:

*And then I call a medium-sized PVS manufacturer and they say: "No, we don't make apps. “[…] They just didn't realize it and of course, the legislator doesn't have to do the work for us now and inform all the PVS manufacturers and message them to implement all this, but it’s important for those who want to make the DiGA project a success to understand: "The PVS manufacturers are sometimes to often not at all helpful to harmful."*

[DiGA manufacturer]

Stepping away from the superordinate perspective and looking at problems and barriers to better use of DiGA within the direct care context, problems surrounding DiGA prescribing physicians, patients, and their interactions were reported. Especially in the early days of DiGA, the interviewed stakeholders reported insufficient knowledge about DiGA, their content, and the corresponding regulations among physicians (DiGA manufacturer, SHI representative, patient representative, physician representative). This was generally acknowledged by physicians while emphasizing the limited capacity for DiGA within a regular workday:

*Today I had another Monday where [I] didn't even know where to stack the patients. And then when some people come in who have problems with their app and need technical support, I find it difficult in some cases how to accommodate that in an open consultation. That might be a bit out of touch with reality.*

[Physician representative]

They also reported heterogeneous interests and skepticism among colleagues and concerns of (partially) being replaced by DiGA at some point (physician representative).

Different stakeholders agreed about insufficient awareness of the information services and content of the DiGA among patients. Patients did not know what DiGA were or how to claim them. The process of going through the SHI fund to receive the activation code hindered patients from using DiGA (DiGA manufacturer, patient representative). Patients also showed low compliance levels according to an SHI representative, which they found out through a company internal survey. There was also concern among patients that the “normal” treatment option may be withheld from them if they opt in to using a DiGA (patient representative).

*And another problem for patients is that they sometimes have the feeling that if they use a DiGA, they will be deprived of another therapy, such as behavioral therapy. So, if you have the feeling that you have to choose either or, then in my experience most patients tend to go down the usual treatment routes.*

[Patient representative]

Stakeholders also expressed similar opinions regarding problems with the prescription process. In some cases, low prescription quality (eg, missing diagnosis) was registered by SHI funds when processing DiGA prescriptions (SHI representative). As mentioned above, the prescription process of DiGA was also criticized, and a digitalized adaptation was called for (DiGA manufacturer). Closely interrelated with the prescription process, the production and distribution of DiGA activation codes were described as problematic in the early days of DiGA. Incorrect codes were sent out, which led to patients not being able to use the DiGA (DiGA manufacturer, SHI representative).

Two stakeholders criticized the missing combination of DiGA use and therapy by physicians. They reported that, especially for risk-associated DiGA (eg, DiGA for mental diseases), monitoring and guided app use might be necessary (SHI representative, patient representative).

*There are also DiGA that are associated with risks, especially when I think of DiGA for depression. This prescription and use should at least be accompanied by a physician. So, I see problems here if the physician basically just prescribes and doesn't stay in conversation with the insured person about how the DiGA actually works.*

[SHI representative]

At the time of the study, DiGA were meant to be used mainly by the patients themselves. Active involvement of physicians was possible, but only a few DiGA incorporated such tools [9]. DiGA manufacturers described the integration of physicians with existing legal regulations as difficult and added that this may cut back on the potential of DiGA (DiGA manufacturer).

## Discussion

### Principal Findings

The aim of this study was to identify problems and barriers regarding the admission, financing, and use of DiGA. As other studies have focused on the apps themselves, we wanted to determine which problems concerning the infrastructure and processes influence optimal use of this new digital health care service. The findings show that some problems can be attributed to the early days of DiGA, while other issues are more prevalent for long-term success. Regarding the admission, the DiGA Fast-Track itself was criticized because of insufficient levels of expertise among the responsible institutions and the duration of the process. From a financial perspective, problems regarding the price negotiations and reimbursement were most frequently discussed. The superior negotiation position of the GKV-SV was pointed out, as well as criticism that reimbursement is independent from DiGA usage. DiGA service provision was criticized on a superordinate level because of, for example, the public perception of these new health care services and negative statements among involved stakeholders. As aforementioned, insufficient knowledge and skills surrounding DiGA were also a problem among patients and health care professionals, along with early issues of the prescription process and a lack of integration of physicians into DiGA use.

### Admission

As mentioned before, some of the identified problems and barriers can be attributed to the short existence of DiGA at the time the survey was conducted (first DiGA: 09/2020, Interviews: 02-06/2022). DiGA manufacturers pointed out the challenges associated with a 3-month certification process, including review and additional paperwork. To this day (July 2025), only 25 applications were rejected after the DiGA Fast-Track process. Notably, 124 applications have been withdrawn from the certification process [34]. As we cannot view all reasons of withdrawal, we can assume that, at least to some extent, it may have been due to deadlines, which were not or could not be met. This leaves the question of whether just the quality of applications of digital health apps is not good enough, or if process adjustments may have the chance of allowing more qualitatively high digital health apps access to the DiGA directory.

In comparison to the 57 currently listed DiGA, over twice as many applications and digital health apps have not been included in the directory. In addition to withdrawals (n=124) and negative decisions (n=25), 14 DiGA have been deleted from the directory after originally being included (total number of DiGA not included: n=163). Again, as the reasons for unsuccessful applications cannot be viewed, this may also suggest that the DiGA Fast-Track with its requirements has the potential to ensure quality and reliability of DiGA [34].

This may be a small indicator that concerns about the DiGA Fast-Track allowing digital health apps to easily enter the German statutory health care market should be examined in more detail. The length of the process was not the only argument for this position. We identified a disagreement about the strength of the requirements of the DiGA Fast-Track as well. While DiGA manufacturers highlighted a high (but bearable) burden for successful completion of the DiGA Fast-Track, especially SHI-related stakeholders mentioned concerns about the (missing) strength of the requirements. Already in the early days of DiGA, the DiGA Fast-Track and its framework (eg, preliminary approved DiGA) were subject to SHI criticism [35]. As laid out above, the high number of withdrawn and negatively decided DiGA applications indicates that existing requirements cannot be met by a large share of applying companies. In addition, the underlying legislation (Digital Health Applications Regulation) has been updated frequently since its introduction in 2021 [36]. It has to be kept in mind that up until the “Act to accelerate the Digitization of the Healthcare System (Digital Act–DigiG; effective since March 2024)”, only DiGA of low-risk classes were reimbursable within SHI. The expansion to higher-risk classes within the act may influence the appropriateness of the DiGA Fast-Track requirements. For example, the act includes the requirement for DiGA of higher risk classes to prove the medical benefit through a prospective comparative study [37].

The preliminary approval of DiGA caused some controversy, as it presents a new and never-before-seen approach to introducing health care services into the SHI system. We like to point out that even if no final results are necessary to obtain preliminary approval, there are still requirements tied to this status. In addition to meeting all “general” requirements (eg, security, functionality, quality, data security), a preliminary systematic data evaluation as well as an evaluation concept is necessary, plausibly showing that the medical benefit can be proven within the preliminary approval period [4]. As this still bears the possibility for the evaluation studies to show the corresponding DiGA does not provide a medical benefit, it opens the door for an early market entry of new, innovative, potentially disruptive technologies. Nevertheless, DiGA are used by patients with diseases. The worst case for any health care services would be harming the patient. To ensure health care services are safe and beneficial to the patient, studies providing evidence of such effects with final, statistically significant information are conducted. These studies must also fulfill high-quality standards. Existing literature shows that some DiGA have deficiencies regarding the quality of the studies showing the medical benefit (for example, lack of blinding, inhomogeneous dropout rates) [38]. Therefore, in the case of DiGA, the balancing of innovation and (final) evidence may currently be prioritizing innovation.

The implementation of admission processes for DiGA-equivalent digital health apps in other European countries (eg, France and Belgium) shows that there is an agreement about DiGA being a necessary part of health care systems in the future. This is underlined by ongoing discussions about the implementation of admission processes in additional European countries, for example, Spain, Austria, and Italy. However, some countries do not prioritize this topic as of now, which cannot be neglected [39].

### Financing

Finally, one of the most talked-about topics in the public discourse surrounding DiGA is their price and the corresponding processes, for example, negotiations between DiGA manufacturers and the National Association of SHI funds. Some of the interviewed experts (eg, physician representatives, SHI representatives) criticized the possibility for DiGA providers to set their own price for the first 12 months of approval. To keep DiGA prices within a reasonable and comparable range, the legislator made adjustments regarding the pricing processes. A collective agreement between the National Association of SHI funds and leading DiGA manufacturer associations was put in place, introducing maximum prices for DiGA, setting the range for the price of the DiGA in relation to other DiGA treating the same or a comparable disease [40].

The opposing views by SHI representatives and DiGA manufacturers regarding the price of DiGA may be due to their competing interests. As the SHI funds are paying for the use of DiGA by their insured persons, lower and criteria-based (eg, quality, usage) prices can lead to lower costs for the corresponding SHI fund. At the same time, DiGA manufacturers are financing (to a significant extent) their company through the income generated from patients using their app, therefore having an interest in higher prices.

### Service Provision

A problem medical providers mentioned was the current prescription process, calling for a more digitalized prescription process (“Digital health solution needs digital prescription” – DiGA manufacturer). This was shared by patients, as some of them did not want to use the DiGA because of the complicated prescription process. With the mandatory introduction of the e-prescription in Germany starting in January 2024, digital prescriptions are now the standard within German health care. They are currently only used for prescribable drugs, but are planned to be extended to DiGA in 2025 [41]. This has the potential of further boosting DiGA as the prescription process and, therefore, the access to DiGA can improve once the e-prescription is established [42]. With problems in the early days of e-prescription now solved, they are mainly seen as an improvement among representatives within the German SHI system [43] and may therefore contribute to more DiGA use when extended. However, even if they are not the main target group of DiGA, the preferences of people with low digital literacy and non–digital natives also need to be incorporated to create an option of low-barrier access to DiGA. This may be of particular relevance for indications that have a substantial number of patients in higher age groups.

A problem was also presented in the low knowledge and skills around DiGA by both the prescribing (medical providers) and the using parties (patients). One tool to tackle this challenge was the DiGA directory, providing a range of information separately presented for health care professionals and patients [9]. Our study showed that for a share of the involved stakeholders, this goal has not been met yet. As growing prescription and usage numbers indicate (see SHI Reports [15,16] for more), we assume the lack of knowledge was—at least to some part—due to the nature of the new digital health service and the introduction during the COVID-19 pandemic. The importance of knowledge and skills regarding digital health care services has also been emphasized by the World Health Organization. Borges do Nascimento et al [44] identified digital health services as possibly “a real asset to the health and care workforce.” To realize these potentials, improving the digital health literacy among health care professionals is a fundamental step.

To further boost DiGA and enable it to thrive, more integration of medical providers in the use of DiGA might be a solution. International Studies have shown that this also has the potential to increase the adherence of the patients [45,46]—which was criticized by SHI representatives—assuring that reimbursed DiGA are being used as good and intended as possible to create a medical benefit for the patient. A large German health insurance fund reported that almost 40% of insured people with prescribed DiGA did not use the app over the intended time span. Of the people who discontinued using the DiGA, only about 20% reported it was due to improved health [47]. To improve adherence and guide the patient, some DiGA have integrated modules to include medical providers [9]. Monitoring of patient data and consultation of the patient at follow-up visits are some examples of these tasks. This appeal results in a call for reimbursement of the corresponding tasks (eg, monitoring of patient data, consulting services) carried out by the medical provider. Up to today, only a few DiGA have negotiated reimbursement for their DiGA-supporting tasks [48]. Applying similar structures to more DiGA may improve adherence among patients and, therefore, the conditions for DiGA to provide a medical benefit to the patient.

However, more integration of health care providers cannot be seen as an isolated option to improve DiGA use. One fundamental component of the long-term success of health care services is the acceptance among medical providers and patients. Since the evaluation reports [15,16] have shown that over 90% of DiGA are prescribed by medical providers, they play a key role as they are (for a large share) the gatekeepers in the distribution of this health care service. At the same time, acceptance of patients is also important, since they are the ones using DiGA. A web-based survey [49] covering potential users in Germany showed that there is a generally high willingness (76%) to use digital health apps. Still, the authors conclude that “targeted communication around effortless usage of mHealth services across age groups and demographics” is necessary to improve digital health app adoption. Another study [50] (covering mental illnesses) adds to this conclusion, for example, outlining that there is unequal access to digital health technologies for some patient groups, and this might lead to the worsening of health inequalities. This emphasizes the importance of addressing the acceptance of medical providers and patients in order to improve digital health app use.

Many of the presented problems show a strong reliance on the German health care system. Nevertheless, the results present an overview of relevant problems for researchers and policymakers from different health care system approaches when considering the implementation of digital health apps. The early days of DiGA contained a wide range of first impressions and statements from stakeholders. As we found out within this study, there was disagreement among involved parties about different aspects of DiGA within the German health care market. There are various reasons why stakeholders potentially disagreed, such as competing interests or system logic. On the one hand, DiGA are brought into the market by DiGA manufacturers, who get reimbursed for their service. On the other hand, as DiGA are part of the statutory health care system in Germany, they are reimbursed by SHI funds. These 2 stakeholders have competing interests, and this showed in opposing statements. One example is the authorization requirements for DiGA, where SHI representatives suggested the criteria are too low, when at the same time DiGA manufacturers described the increasing requirements as challenging (refer to Results: Admission). Therefore, it has to be kept in mind that some of these contradictory statements are also influenced by interests and system logic as described above.

Nevertheless, DiGA made their start in 2020 and have been growing since. As laid out throughout this article, this was accompanied by challenges along the way, not unexpectedly due to the iterative nature of the legislative process. On the side of the stakeholders involved in the actual distribution of DiGA, many challenges can be attributed to the early days. These aspects, therefore, should be addressed early on when implementing a DiGA-equivalent health care service in other countries.

### Limitations

First, we interviewed stakeholders from relevant stakeholder groups. Nevertheless, within each stakeholder group, we could not completely ensure representativeness; for example, we did not interview a DiGA manufacturer for each disease in the DiGA directory. The sample is also not representative of factors like the number of insured people with the SHI funds or the share of the corresponding DiGA indication among all listed DiGA. In order to still cover a large share of the involved stakeholders, we included central associations of the corresponding stakeholder groups (eg, GKV-SV, DiGA-Spitzenverband [DiGA-SV]). Even though we tried to cover all relevant stakeholder groups by sending out multiple requests, some declined participation or opted for later involvement in the research project, for example, BfArM. We also only included one patient representative, which may lead readers to think this important stakeholder group is underrepresented in our study. We conducted a focus group and interview study exclusively covering the patient’s perspective prior to this study and therefore refer to the corresponding article for more insights into patients’ views [28].

We want to point out that all statements made are subjective and only represent the view of the interviewed stakeholder and may or may not translate to DiGA in general. The roles of the participants or the stakeholder group, which they represented, and the corresponding statements may have been influenced by personal interests or a political agenda. Statements were checked with existing evidence but remain subjective to some extent. Since this research paper aims to report problems surrounding DiGA from different stakeholder perspectives, this limitation has to be accepted in order to reach the research goal.

When conducting an interview study, the role and position of the involved researchers is an aspect that can generally influence the results of the study (eg, personal preconceptions regarding the research topic, prior collaboration between researchers and interview participants). To minimize the influence of this limitation, all steps of the study were generally carried out by at least 2 researchers. The generated data was coded and analyzed by 2 researchers independently, with subsequent cross-checking by a third experienced senior researcher. In addition, all transcripts and codes were quality assured by a researcher not primarily involved in the corresponding interview in order to identify any analytical results and conclusions influenced by preconceptions.

Another limitation is the period of time during which we conducted the interviews. Since we started conducting the interviews in February 2022, there have been only a few DiGA inducted into the DiGA directory. This ultimately led to a few prescribed DiGA, which decreased the representativeness of the generated evidence. To account for this limitation, we laid out which of the presented problems have already been solved and how processes and the general perception of DiGA may have improved since then. Still, we cannot fully guarantee that some of the aspects we described may be transitional or only related to early implementation efforts and may have been resolved after the study period. At the same time, we assume that some of the problems, for example, the imbalance between stakeholders during price negotiations or the different perceptions toward the preliminary approval of DiGA, may not be transitional and still have an influence on the current DiGA landscape.

The online format (interview via videocall) may have also impacted the quality of the generated data, as it was not possible to control, for example, for additional people in the room or technical problems. However, since the interviews were conducted almost 2 years after the COVID-19 pandemic started, online video tools were already common. There were also no major technical problems during the interviews. There is research indicating that there are no significant differences between online and face-to-face interviews [51,52].

### Conclusions

With the introduction of DiGA to the German health care system, a step toward more digitization was taken, with the potential of improving care for patients. Nevertheless, there was also skepticism and risks to how well the new technology would improve and fit into the German health care system. The identified problems can be seen as “necessary” steps in the development and evolution of DiGA, for example, early problems with activation codes, insufficient knowledge about DiGA among involved actors, and different perceptions about the prices of DiGA. Some of the barriers have already been addressed along the way, for example, different adaptions with pricing of DiGA. This goes along with new health policies further expanding DiGA to higher-risk classes, which opens up opportunities for new DiGA and, therefore, more patients to benefit from it. This also shows in a higher number of DiGA being prescribed and more DiGA being added to the DiGA directory.

Therefore, we are concluding that problems and barriers have been and are still impacting DiGA on the way to becoming a contributing component of patient care. To identify the current state of DiGA and which problems may have been solved and additionally arisen through the years, new research investigating the perspective of a similar group of stakeholders may be necessary. We also want to point out that although we aimed at identifying problems and barriers in the early days of German DiGA use, one result of our study was that a large share of the questioned stakeholders had a positive attitude toward the potential of DiGA and the processes in the early implementation phase. Yet, as our study has shown, there is still room for improvement so that DiGA can reach its proclaimed potential.

Finally, the identified problems and barriers can help other countries when implementing DiGA-equivalent digital health apps. With conclusions drawn from the presented challenges, an optimized implementation process may lead to an early realization of positive effects.

## Supplementary material

10.2196/73332 Multimedia Appendix 1 Coding scheme.

10.2196/73332 Checklist 1 COREQ checklist.

## Abbreviations

BfArM: Bundesinstitut für Arzneimittel und Medizinprodukte (Federal Institute for Drugs and Medical Devices)
COREQ: Consolidated Criteria for Reporting Qualitative Studies
DiGA: Digitale Gesundheitsanwendung (digital health application)
GKV-SV: GK-Spitzenverband (National Association of Statutory Health Insurance Funds)
mHealth: mobile health
SHI: statutory health insurance
