# Supplementary material for: Problems and Barriers Regarding the Admission, Financing, and Service Provision of Digital Health Apps: Qualitative Stakeholder Survey
Source: J Med Internet Res. 2026 Feb 9;28:e73332. doi: 10.2196/73332 (PMC12885185; doi:10.2196/73332)
Supplement: Multimedia Appendix 1 [file jmir-v28-e73332-s001.docx]

# Problems and Barriers regarding the Certification, Financing and Use of Mobile Health Applications: A Qualitative Stakeholder Survey

**Multimedia Appendix 1:** Coding Scheme

The coding scheme consists of deductive and inductive codes [see Mayring (2015) and Kuckartz (2018); full references in main article]. Inductive codes are marked with: (i)

1. **General Problems**
   1. Current institutional and process-related problems (i)
   2. Negative public reporting (i)
   3. Transparency (i)
2. **Problems:** *Admission*
   1. Problems with current admission process (i)
   2. Problems with evidence (i)
   3. Preliminary approval (i)
   4. Immediate market entry (i)
3. **Problems:** *Finance*
   1. Alternative financing options (i)
   2. Price negotiations (i)
   3. Payment for DiGA (i)
   4. Financing of DiGA-manufacturers (i)
4. **Problems:** *Healthcare Service Provision*
   1. Prescription Process (i)
5. **Problems:** *Statutory Health Insurance*
   1. Price expectations (i)
   2. Uncooperative actions (i)
   3. Attitude (i)
   4. DiGA-related processes (i)
6. **Problems:** *Association of Statutory Health Insurance (GKV-Spitzenverband (GKV-SV))*
7. **Problems:** *DiGA-manufacturers*
   1. Processes (i)
   2. Approach of DiGA manufacturers (i)
   3. Requirements for DiGA manufacturers (i)
   4. Test-Accounts (i)
8. **Problems:** *Patients*
   1. Knowledge (i)
   2. Attitude (i)
   3. Usage (i)
9. **Problems:** *Healthcare Service Providers*
   1. Knowledge (i)
   2. Prescription (i)
   3. Attitude (i)
10. **Problems:** *Federal Institute of Drugs and Medical Devices (Bundesinstitut für Arzneimittel und Medizinprodukte (BfArM))*
    1. Duration Fast-Track Process (i)
11. **Problems:** *Federal Institute for Information Security (Bundesinstitut für Sicherheit in der Informationstechnik (BSI)) (i)*
12. **Problems:** *Practice management system manufacturer (Praxisverwaltungssystem-Hersteller (PVS)) (i)*
13. **Problems:** *Notified Bodies (Benannte Stellen) (i)*
